# Nejire/dCBP-mediated histone H3 acetylation during spermatogenesis is essential for male fertility in *Drosophila melanogaster*

**DOI:** 10.1371/journal.pone.0203622

**Published:** 2018-09-07

**Authors:** Tim Hundertmark, Stefanie M. K. Gärtner, Christina Rathke, Renate Renkawitz-Pohl

**Affiliations:** Philipps University of Marburg, Department of Biology, Developmental Biology, Marburg, Germany; Texas A&M University, UNITED STATES

## Abstract

Spermatogenesis in many species including *Drosophila melanogaster* is accompanied by major reorganisation of chromatin in post-meiotic stages, involving a nearly genome-wide displacement of histones by protamines, Mst77F and Protamine-like 99C. A proposed prerequisite for the histone-to-protamine transition is massive histone H4 hyper-acetylation prior to the switch. Here, we investigated the pattern of histone H3 lysine acetylation and general lysine crotonylation in *D*. *melanogaster* spermiogenesis to elucidate a possible role of these marks in chromatin reorganisation. Lysine crotonylation was strongest prior to remodelling and the deposition of this mark depended on the acetylation status of the spermatid chromatin. In contrast to H4 acetylation, individual H3 acetylation marks displayed surprisingly distinct patterns during the histone-to-protamine transition. We observed that Nejire, a histone acetyl transferase, is expressed during the time of histone-to-protamine transition. Nejire knock down led to strongly reduced fertility, which correlated with misshaped spermatid nuclei and a lack of mature sperm. *protA* and *prtl99C* transcript levels were reduced after knocking down Nejire. ProtB-eGFP, Mst77F-eGFP and Prtl99C-eGFP were synthesized at the late canoe stage, while histones were often not detectable. However, in some cysts histones persist in parallel to protamines. Therefore, we hypothesize that complete histone removal requires multiple histone modifications besides H3K18ac and H3K27ac. In summary, H3K18 and H3K27 acetylation during *Drosophila* spermatogenesis is dependent on Nejire or a yet uncharacterized acetyl transferase. We show that Nejire is required for male fertility since Nejire contributes to efficient transcription of *protA* and *prtl99C*, but not *Mst77F*, in spermatocytes, and to maturation of sperm.

## Introduction

One of the most dramatic processes of chromatin remodelling occurs during the post-meiotic phase of spermatogenesis. The previously histone-based chromatin becomes a highly compact structure due to the nearly genome-wide replacement of histones, first by transition proteins, which are then replaced by small basic proteins called protamines. The general aspects and other characteristic features of this large-scale chromatin compaction are conserved from fly to man (reviewed in [1–4]. However, the molecular processes that finally lead to histone replacement and chromatin condensation remain largely unknown.

One mechanism proposed to be a prerequisite for the histone-to-protamine transition is a genome-wide hyper-acetylation of histones occurring in the total absence of transcription (Braun 2001; Sassone-Corsi 2002). In mouse and *D*. *melanogaster*, hyper-acetylation of histone H4 is observed in elongating spermatids and gradually disappears in parallel with histone degradation [5–7]. A lack of histone hyper-acetylation correlates with male infertility in mouse and man [8, 9]. Furthermore, histones lack hyper-acetylation in species where histones are not replaced by protamines [10]. Similarly, histone hyper-acetylation is essential for chromatin reorganisation in *D*. *melanogaster* spermiogenesis, since inhibition of acetylation after the pre-meiotic transcriptional phase blocks further spermatid differentiation, and the chromatin remains histone bound (Awe and Renkawitz-Pohl, 2010). These data clearly demonstrate that hyper-acetylation is tightly linked to histone replacement. Simultaneously with H4 hyper-acetylation, H3K9 acetylation, lysine crotonylation and H3K4, H3K9 and H3K79 methylation are described to increase prior to the transition from histones to protamines [7, 11–13]. As in mammals, in *D*. *melanogaster* histones are successively replaced by protamines (ProtA and ProtB) and in *Drosophila* by two additional abundant sperm chromatin components, Mst77F [14–17] and Protamine-like99C (Prtl99C) [18].

How H4 hyper-acetylation and other histone modifications regulate the histone-to-protamine transition in elongating spermatids is poorly understood. According to the histone code hypothesis, modifications could define specific signals and serve as an interface language between histones and chromatin modifying activities [19]. As part of the histone code, acetylated lysine residues, as well as other post-translational histone modifications, provide binding sites for specific protein interactions. Since both the assembly and removal of histones in somatic cells during replication, DNA repair and transcription involve specialized machineries (reviewed in [20], histone hyper-acetylation by distinct histone acetyl-transferases in elongating spermatids could serve as a signal to recruit the machinery displacing histones. Part of such a machinery could be bromodomain-containing proteins, since the bromodomain is a motif that specifically binds to acetylated lysine residues [21] (for review see [22]). In *D*. *melanogaster*, blocking histone acetylation specifically in the post-meiotic phase by inhibitors led to loss of protamine deposition. As in mice, *D*. *melanogaster* histone hyper-acetylation cannot be the sole inducer of the switch since premature histone hyper-acetylation does not lead to a premature protamine-based chromatin structure [23].

Here, we identified post-translational histone modifications accompanying histone-to-protamine transition in *D*. *melanogaster* with the focus on histone H3 acetylation. In addition, we searched for enzymes mediating histone modifications, such as histone acetyl-transferases, which may function as “writer factors” in the histone-protamine-transition phase. We show that histone H3 acetylation displays a highly diverse pattern compared to H4 acetylation. Remarkably, after meiosis H3K18ac and H3K27ac are limited to the early canoe stage just prior to the histone-to-protamine transition. We postulate Nejire or an enzyme with the same specificity as a putative “writer” of these post-meiotic H3 histone acetylations. Germ line-specific knock down of Nejire led to a lack of mature sperm, demonstrating a function of Nejire in proper spermatid differentiation.

## Materials and methods

### Fly stock and maintenance

*D*. *melanogaster* strains were maintained on standard medium at 25°C. *w*^*1118*^ was used as the wild-type strain. Protamine B-mCherry (ProtB-mCherry) transgenic flies were generated by modifying the ProtamineB-eGFP construct of [15]. For *nejire* functional studies the RNAi line v105115 (Vienna Drosophila Resource Center) was combined with ProtB-GFP, Mst77F-eGFP [15] and Prtl99C-eGFP [18] transgenic fly lines. For the *nejire* knock down, v105115 protB-GFP males were crossed against virgin females of bam-Gal4/bam-Gal4; Sp/CyO; bam-Gal4-VP16/MKRS. Crossings for knock down experiments were maintained at 30°C.

### Culture of pupal testes and inhibitor treatment

Pupal testes cultures of transgenic flies carrying ProtB-mCherry were established as described in [23]. In short, pupal testes were dissected in Shields and Sang M3 insect culture medium (Sigma- Aldrich Cat#S8398) supplemented with 10% foetal bovine serum (heat inactivated, insect culture tested, Sigma-Aldrich Cat#F3018), 100 U/ml penicillin and 100 mg/ml streptomycin (Gibco-Invitrogen Cat No. 15140–148). The intact testes were transferred to a 25-well plate. For inhibitor treatment, generally, six pupal testes were used for each inhibitor and control per experiment. The experiments were repeated at least three times.

Testes were treated with anacardic acid (AA, Merck Biosciences; 28.69 mM in DMSO stock) and trichostatin A (Cell Signalling Tech.; 4 mM in ethanol stock) in appropriate dilution in culture medium. Control cultures with solvent alone were analysed in parallel. Cultures were incubated for 24 h at 25°C.

#### Sterility tests

One adult male of each genotype was placed together with two wild-type virgin females in a vial for 3 days at 25°C. After 3 days, the parental generation was removed from the vials. After 2 weeks, offspring in each vial were counted.

### Antibodies and immunofluorescence staining

For immunofluorescence analysis, adult testes and cultured pupal testes of transgenic flies carrying ProtB-mCherry were squashed and treated essentially as described in Hime et al.[24]. DNA was stained with Hoechst 33258 dye. H3K4ac was probed with Histone H3K4ac antibody (pAb) (active motif, Cat. No. 39382) in 1:100, 1:250, 1:500 and 1:1000 dilutions. Histone H3K9ac was detected with antibody anti-Acetyl-Histone H3 (Ac-Lys9) (Sigma, Cat. No. H9286) at a 1:500 dilution. H3K14ac was detected with Histone H3K14ac antibody (pAb) (active motif, Cat. No. 39698) at a 1:500 dilution. H3K4ac was detected with Histone H3K4ac antibody (pAb) (Millipore) at 1:100, 1:250, 1:500 and 1:1000 dilutions. H3K18ac was detected with Histone H3K18ac antibody (pAb) (active motif, Cat. No. 39756) at a 1:500 dilution. H3K23ac was detected with antibody Histone H3K23ac antibody (pAb) (active motif, Cat. No. 39132) at a 1:600 dilution. H3K27ac was detected with Histone H3K27ac antibody (pAb) (active motif, Cat. No. 39136) at a 1:500 dilution. H3K36ac was detected with Histone H3K36ac antibody (pAb) (active motif, Cat. No. 39380) at a 1:250 dilution. H3K56ac was detected with anti-acetyl-Histone H3 (Lys56) antibody (Millipore, 04–1135) at a 1:1000 dilution. H3K64ac was detected with Histone H3K64ac antibody (pAb) (active motif, Cat. No. 39546) at 1:100, 1:250, 1:500 and 1:1000 dilutions. H4K5ac was detected with Histone H4K5ac antibody (pAb) (active motif, Cat. No. 39170) at a 1:500 dilution. H4K8ac was detected with Histone H4K8ac antibody (pAb) (active motif, Cat. No. 61103) at a 1:500 dilution. H4K12ac was detected with antibody Histone H4K12ac antibody (pAb) (active motif, Cat. No. 39928) at a 1:500 dilution. Kcr was detected with antibody anti-crotonyllysine antibody (PTM Biolabs, Cat.No. PTM-501) at 1:200 dilution. Histones were detected with antibody anti-Histone, F152.C25.WJJ (Millipore) at a 1:1200 dilution. Nejire was detected with antibody anti-CBP [25] at a 1:1000 dilution. These primary antibodies were visualized with anti-rabbit, DyLight488-conjugated secondary antibody (Vector Laboratories) at 1:100 dilutions.

### RNA isolation and qPCR

RNA from 300 Bam>>v105115 testes or v105115 undriven testes was extracted using TRIzol (Invitrogen). Extracted total RNA was treated with RNase-free Turbo DNase (Ambion) and purified using an RNeasy mini kit (Qiagen). 1 μg of DNAse treated and purified RNA was used for cDNA synthesis with the Transcriptor First Strand cDNA Synthesis kit (Roche) according to the manual in a 20 μl reaction. qPCR reactions were set up with 10 μl iTaq™ Universal SYBR® Green Supermix (Bio-Rad), 4 μl ddH_2_O, 0.5 μl (10 μM) gene-specific primer A, 0.5 μl (10 μM) gene-specific primer B and 5 μl cDNA (1:25 dilution). qPCR was performed in three technical replicates on the Mx3000P qPCR. Ct values were normalized to the mRNA expression level of Rpl32. Mean value and SEM of three biological replicates was calculated using GraphPad PRISM version 5.03. For statistical analysis one-sample t-test with a hypothetical value of 1 was used.

## Results

### Multiple histone H3 acetylations in early and late canoe stages of the histone-to-protamine transition phase of *D*. *melanogaster* spermatogenesis

In *D*. *melanogaster*, the histone-to-protamine transition takes place during the so-called canoe stage. Here, the early canoe stage is defined by the start of histone removal, and the late canoe stage by the start of protamine accumulation [7]. Hyper-acetylation of histone H4 is proposed to be a prerequisite for histone removal during spermiogenesis in flies and mammals. In addition, a simultaneous increase in histone H3 acetylation was shown in mammals [6]. However, little is known about a possible role of H3 acetylation in post-meiotic chromatin remodelling.

**Fig 1:**
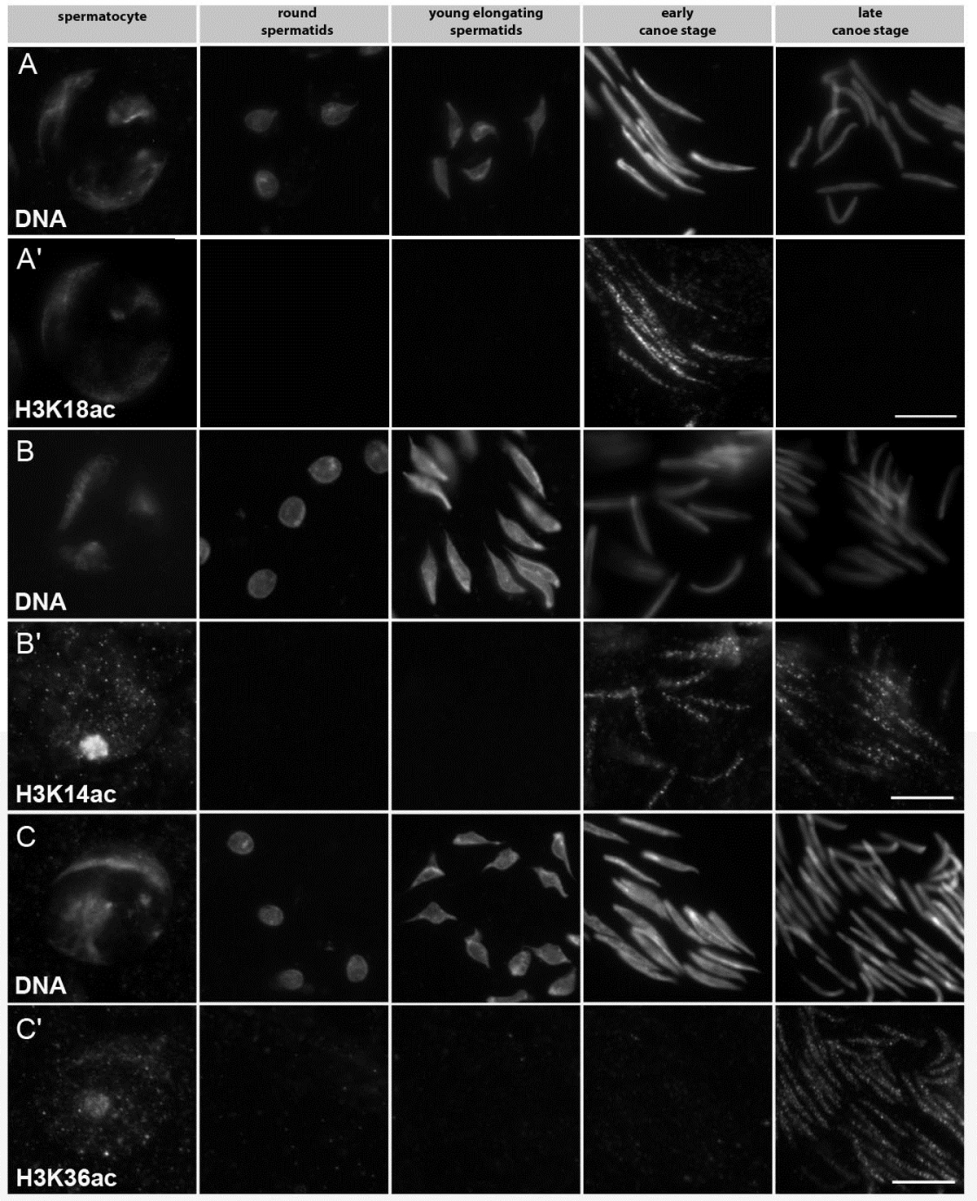
Histone H3 lysine acetylation displays a variable pattern in *Drosophila* spermiogenesis. (A-Cʹ) Immunostaining of squashed preparations of spermatocyte and spermatid nuclei from adult testes of ProtB-mCherry expressing flies. (A, B, C) DNA visualized with Hoechst dye. Expression of the ProtB-mCherry fusion protein (from late canoe stage onward) was used to distinguish early and late canoe stage spermatids (not shown). (Aʹ) H3K18ac detected with an anti-H3K18ac antibody was present prior to the switch from histones to protamines in early canoe stage spermatids in a speckled pattern at the nuclei (column 4). (Bʹ) H3K14ac detected with an anti-H3K14ac antibody in early and late canoe stage spermatids gives a speckled pattern at the nuclei (column 4). (Cʹ) H3K36ac detected with an anti-H3K36ac antibody gives a speckled pattern at the nuclei of late canoe stage spermatids (column 5). Scale bar: 10 μm.

Histone H4 acetylation is continuously visible from the spermatocyte stage to the canoe stage, where H4 hyper-acetylation is evident. In contrast, the histone H3 acetylation displayed an unexpected specific pattern within the stages of spermiogenesis (Table 1). We aimed to identify the overall pattern of histone H3 acetylation in spermiogenesis of *D*. *melanogaster* using specific commercially available antibodies against acetylated lysine residues K4, K9, K14, K18, K23 and K27 in the N-terminal domain of histone H3 and K36, K56 and K64 in the globular core domain (summarized in Table 1). Of these antibodies anti-H3K4ac and anti-H3K64ac showed no specific signal in germ cells. H3K56 acetylation was only detectable in primary spermatocytes (data not shown). H3K27ac is present in spermatocytes and prominent later in early canoe stage nuclei (Table 1). In addition to acetylation in primary spermatocytes, the other antibodies displayed characteristic post-meiotic acetylation patterns that for the most part allowed us to group post-meiotic histone H3 lysine acetylation into three classes: (1) acetylation that occurs prior to the histone to protamine transition; (2) acetylation occurring during the time of the transition (late in the early canoe, and beginning of late canoe stage), and (3) acetylation that occurs when most of the chromatin is protamine based (summarized in Table 1; example shown in Fig 1).

**Table 1:** Acetylation and crotonylation of histones in nuclei at different stages of spermatogenesis.

	Spermatocytes	Round spermatid	Youngelongatingspermatid	Early canoe stagespermatid	Late canoe stagespermatid	Individualizedsperm
Anti-H4K5ac	+	+	+	+	-	-
Anti-H4K8ac	+	+	+	+	-	-
Anti-H4K12ac	+	+	+	+	-	-
Anti-H4K16ac	-	-	-	-	-	-
Anti-H3K4ac	-	-	-	-	-	-
Anti-H3K9ac	+	-	+	+	-	-
Anti-H3K14ac	+	-	-	+	+	-
Anti-H3K18ac	+	-	-	+	-	-
Anti-H3K23ac	+	-	-	+	+	-
Anti-H3K27ac	+	-	-	+	-	-
Anti-H3K36ac	+	-	-	-	+	-
Anti-H3K56ac	+	-	-	-	-	-
Anti-H3K64ac	-	-	-	-	-	-
Anti-Kcr	+	-	-	+	(+)	-

Results are based on immunostaining of squashed testes preparations. Note that samples were analysed in a qualitative manner: +, detectable signals; -, no detectable signals; (+), rarely detectable signals. See the text for explanation of the colours.

In the first group, histone H3K9, H3K18 and H3K27 acetylation (Fig 1Aʹ) were detected prior to transition (Table 1, green). H3K9ac was strongest in young elongating and early canoe stage nuclei (Table 1). The signal disappeared in conjunction with histone degradation. H3K18ac (Fig 1Aʹ) and H3K27ac (Table 1, green) are remarkable because of their speckled patterns, which occurred only in early canoe stage nuclei.

The second group comprises H3K14ac (Fig 1Bʹ) and H3K23ac (Table 1, blue); both showed a speckled pattern in early and late canoe stage nuclei. H3K36ac represents a third class, since it was exclusively found in late canoe stage nuclei, clearly visible as a speckled pattern (Fig 1Cʹ and Table 1, orange).

### The histone mark lysine crotonylation is detectable in spermatocytes and canoe stage nuclei

**Fig 2:**
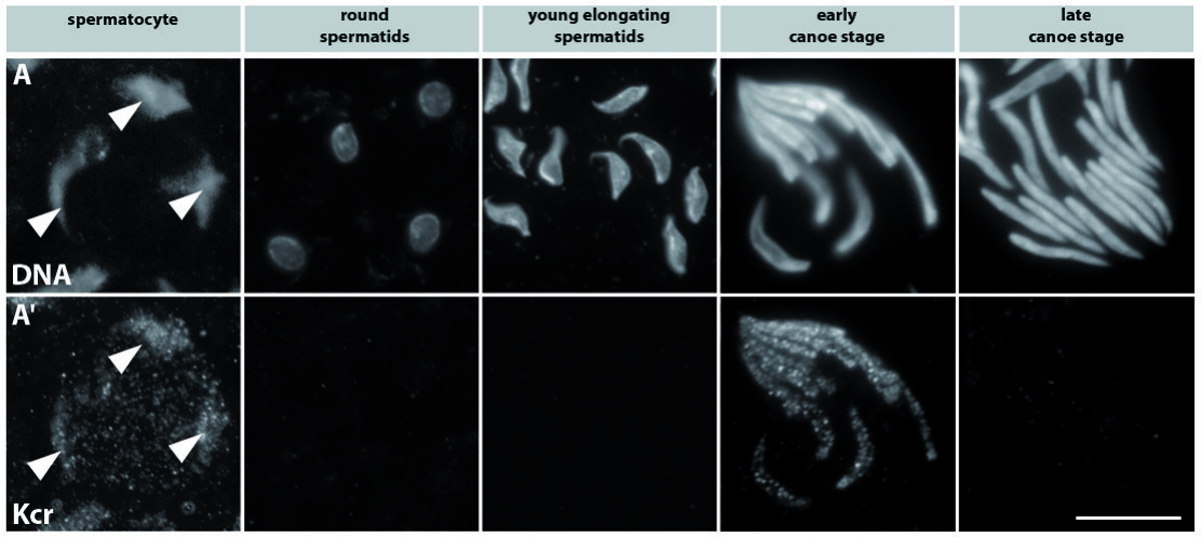
Histone lysines are crotonylated mainly at the early canoe stage shortly before protamine deposition in post-meiotic spermatid nuclei. Immunostaining of squashed testes preparations from ProtB-mCherry expressing flies. Expression of the ProtB-mCherry fusion protein was used to distinguish early and late canoe stage spermatids (not shown). (A) DNA visualized with Hoechst dye. (Aʹ) Lysine-crotonylation detected with an anti-Kcr antibody over the chromosomes in primary spermatocytes (column 1, arrowheads). In early canoe stage nuclei the Kcr signal reappeared and remained until ProtB-mCherry expression had just started in late canoe stage spermatids (column 4–5). Scale bar: 10 μm.

In mouse, a recently discovered new histone mark, lysine crotonylation (Kcr), was reported to show intense labelling during elongating spermatid steps 9–11 ([13] for review see [26]). The intense lysine crotonylation signal coincides with the genome-wide histone hyper-acetylation prior to the switch from histones to protamines shown by [6]. Since lysine crotonylation is an evolutionarily conserved histone mark in *D*. *melanogaster* and mouse, we investigated the distribution of this histone modification in *Drosophila* spermatogenesis by staining squashed preparations of adult testes using an anti-Kcr antibody. Kcr is detectable in primary spermatocyte nuclei over the chromosome regions (Fig 2, column 1, arrowheads). In post-meiotic stages, Kcr is restricted to canoe stage nuclei where the speckled signal is most prominent in early canoe stage nuclei (Fig 2, column 2; Table 1). Thus, we conclude that Kcr disappears in correlation with histone degradation.

### Anacardic acid blocks H3 acetylation and lysine crotonylation whereas TSA induces premature H3 acetylation and lysine crotonylation at the round nuclei stage

As we have shown previously, histone acetylation is essential for the transition from histone-based chromatin to protamine-based chromatin in *D*. *melanogaster* spermiogenesis [23]. To gain further insights into the role of H3 acetylation and lysine crotonylation during the time of transition, we used our established culture system for pupal *Drosophila* testes monitored with RFP or GFP tagged protamines [27]. In these cultures, the histone-to-protamine switch takes place as within the intact fly. The time of transition is easily assessed since the first spermatid cysts switch to protamine-based chromatin between 24 and 48 hours after puparium formation (APF). These properties make cultured pupal testes a valuable test system to study the histone to protamine transition using inhibitor treatment.

To study the effect of inhibiting histone acetyl-transferase (HAT) and histone deacetylase (HDAC) on H3 lysine acetylation and lysine crotonylation specifically in the post-meiotic phase, we isolated pupal testes of ProtB-mCherry transgenic flies at around 24 h APF. To block histone acetylation, these testes were subjected in culture to 150 μM of the HAT inhibitor anacardic acid (AA) for about 24 hours. A known inhibitor of several HAT families [28]. AA neither induces apoptosis nor shows cytotoxic effects in cultured testes, but leads to a block in elongating spermatid differentiation [23]. To block HDAC activity we treated cultured testes for 24 h with 50 μM trichostatin A (TSA). TSA inhibits both class I and II HDACs [29, 30] leading to premature H4 acetylation in *Drosophila* spermiogenesis, while showing no obvious effect on histone to protamine transition [23].

**Fig 3:**
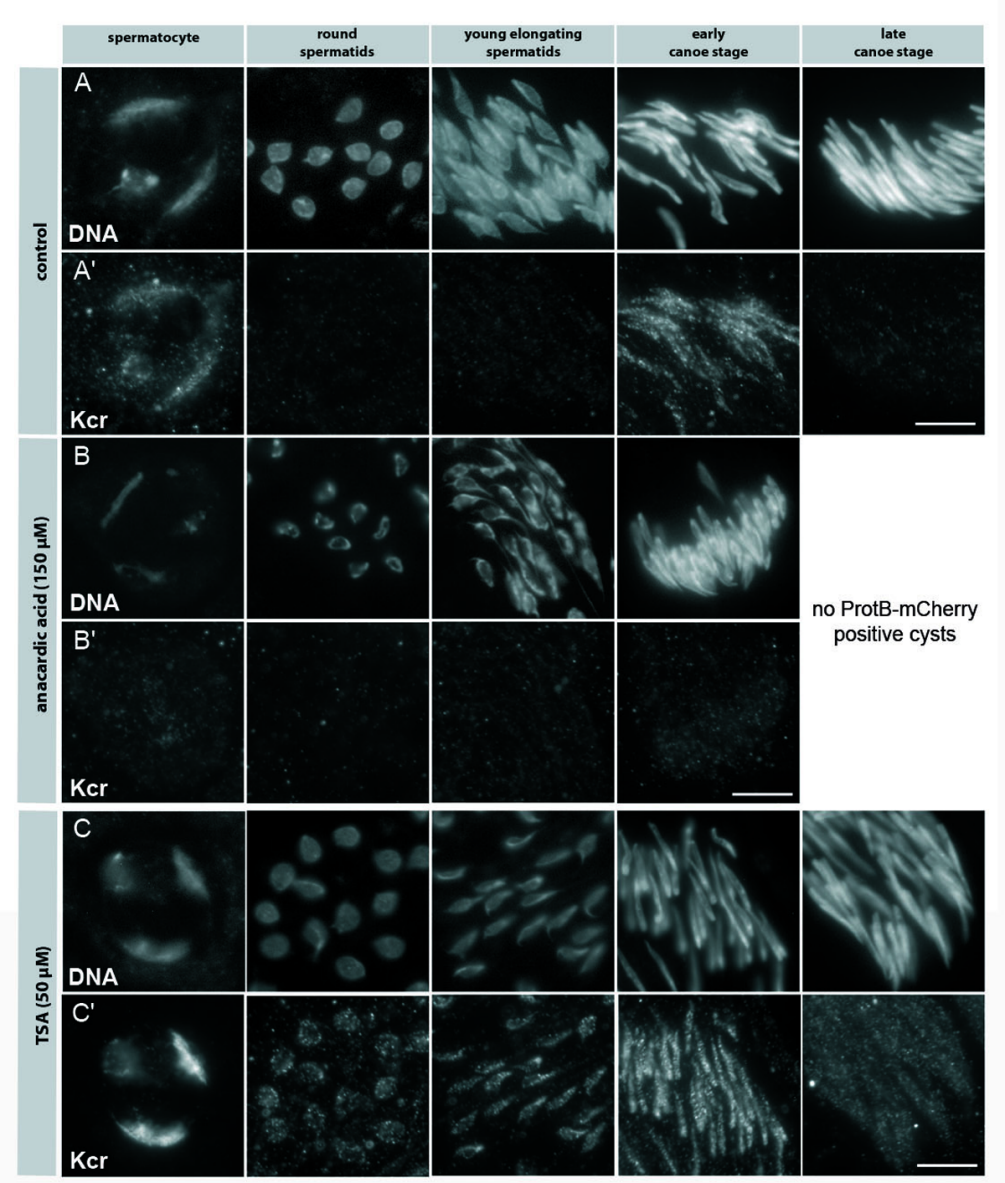
Histone lysine crotonylation depends on histone acetylation. Squashed preparations of spermatid nuclei from pupal testes of ProtB-mCherry expressing flies at 24 h APF incubated for 24 h with either 150 μM AA (B, Bʹ) and 50 μM TSA (C, Cʹ) compared to untreated testes (A, Aʹ). (A, B, C) DNA visualized with Hoechst dye. (Aʹ, Bʹ, Cʹ) Kcr detected with an anti-Kcr antibody. Expression of the ProtB-mCherry fusion protein was used to distinguish early and late canoe stage spermatids (not shown). Treatment with AA led to a complete loss of Kcr (Bʹ) compared to untreated testes (B). In addition, further development of spermatids was blocked (Bʹ). Treatment with TSA (C, Cʹ) led to a strong increase in Kcr at the chromosomal regions in primary spermatocytes (Cʹ, column 1). In post-meiotic stages, premature Kcr occurred in round and young elongating spermatid nuclei (Cʹ, columns 2 and 3). Scale bar: 10 μm.

Since the behaviour of lysine crotonylation with respect to HATs and HDACs has only been described in cell culture experiments [13] we examined Kcr distribution after AA and TSA treatment in *D*. *melanogaster* spermatogenesis. In control testes, Kcr was visible at the early canoe stage up until the late canoe stage was reached (Fig 3Aʹ). At this stage protamines are also deposited in cultured testes, as monitored by ProtB-mCherry expressing post-transition spermatids, which were detectable after 24 h of incubation (data not shown).

In testes treated with AA no late canoe stage spermatids had developed (Fig 3B and 3Bʹ). As expected from our studies with H4 acetylation, both H3K14ac and H3K18ac are no longer detectable in AA treated testes (data not shown). For adding and removing Kcr on histones it has been suggested that the enzymes responsible are independent from those mediating lysine acetylation and deacetylation [13]. Thus, we expected no changes in Kcr distribution upon treatment with AA and TSA. Surprisingly, inhibition of HATs led to a complete loss of Kcr in cultured testes (Fig 3Bʹ) compared to control testes (Fig 3Aʹ). TSA treatment increased acetylation at all stages where HDAC activity is required (not shown) and resulted in enhanced and premature Kcr addition in round and young elongating spermatids (Fig 3Cʹ).

In summary, our data further strengthen the role of histone acetylation as being an essential feature in allowing *D*. *melanogaster* spermatids to develop further, including the transition from histone- to protamine-based chromatin. We identified Kcr as a new histone mark that depends on the acetylation status of the spermatid chromatin and probably plays a role during *D*. *melanogaster* spermiogenesis.

### The histone acetylase Nejire/dCBP is detected in spermatocytes and in the late canoe stage

**Fig 4:**
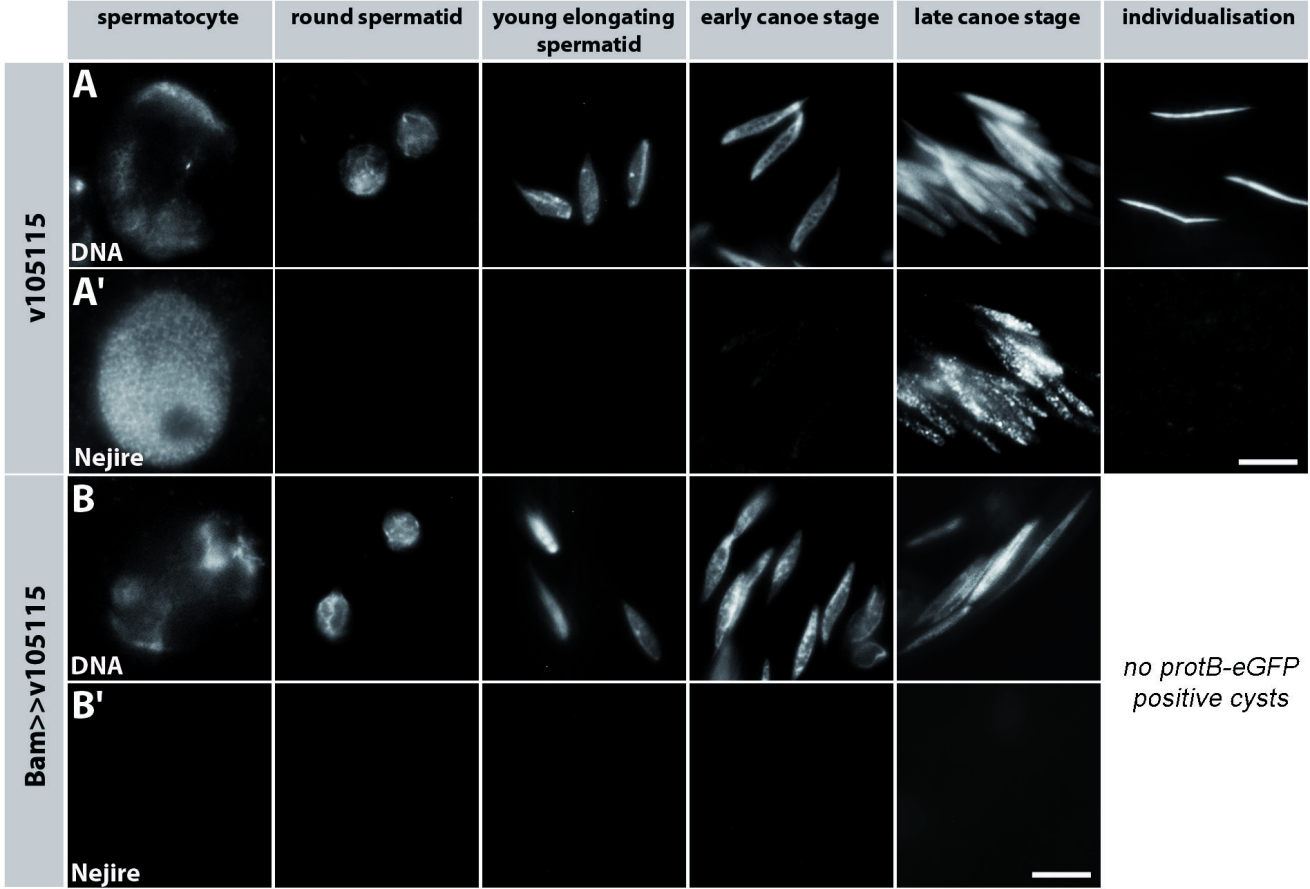
Nejire/dCBP is stage specifically expressed in spermatocytes and spermatid nuclei. Immunostaining of squashed testes preparations from ProtB-mCherry transgenic flies. Expression of the fusion protein was used to distinguish early and late canoe stage spermatids (not shown). (Aʹ) Nejire/dCBP was detected with an anti-dCBP antibody in nuclei of spermatocytes and spermatids, and (A) DNA visualized with Hoechst dye; (column 3–5). Bʹ) Nejire/dCBP is efficiently knocked down by RNAi in spermatocytes in spermatocyte nuclei (column 1) and in late canoe stage spermatids (column 4); (B) DNA was visualized with Hoechst dye. Scale bar: 5 μm.

The extensive reorganisation of chromatin during spermiogenesis requires stage-specific patterns of histone lysine acetylation. Consequently, we searched for enzymes that set these marks and thereby play a putative role in chromatin remodelling and spermatogenesis. H3K18ac and H3K27ac are specifically detected in the early canoe stage (Table 1). These are marks set by the CREB-binding protein (CBP), a lysine acetyl transferase also known as Nejire/dCBP in *Drosophila* [31]. Therefore, we concentrated on analysing Nejire/dCBP. Squashed testes preparations stained with an antibody against Nejire/dCBP revealed expression of Nejire/dCBP in somatic cells of the testes (data not shown) and in the germ line (Fig 4Aʹ). Nejire/dCBP was expressed in primary spermatocytes, then not detected in early post-meiotic stages, but reappeared in late canoe stage nuclei (Fig 4Aʹ). Lysine acetylation mediated by Nejire/dCBP, i.e. H3K18ac and H3K27ac, was detected at the early canoe stage (Table 1). This seemed a contradiction to the visibility of Nejire/dCBP at the late canoe stage, were histones are not detectable anymore. We searched in FlyBase for other candidates with the ability to acetylate H3K18 and H3K27. The genome of *D*. *melanogaster* contains numerous genes with coding capacity for acetyl transferases. Corresponding transcripts were found in testes of adult males for many of them. For some of these acetyl transferases, the targeted histone or a defined lysine residue is known, but none of them was characterized as targeting H3K18 and H3K27 (S1 Table). Thus, we considered that Nejire/dCBP might be the responsible enzyme and only detectable in late canoe stage because Nejire/dCBP is present in chromatin remodelling complexes and not accessible to the antibody during the early canoe stage, while the N-terminal acetylation marks H3K18ac and H3K27ac are accessible to the antibody. At the late canoe stage, histones are not detectable anymore in the already highly condensed chromatin, thus at this time Nejire likely is in the nucleoplasm and therefore might accessible to the antibody at this time. Thus, we approached the role of Nejire/dCBP both by knock down specifically in the male germ line and by specifically inhibiting acetylation during spermiogenesis.

### Despite Nejire knock down Prot-eGFP, Mst77F-eGFP and Prtl99C-eGFP are synthesized

Inhibition of all acetylations by inhibitors led to the failure of protamination. Therefore, we asked whether knocking down Nejire/dCBP dependent H3 acetylation is sufficient to abolish protamination.

To obtain a functional insight into the *in vivo* role of Nejire/dCBP in protamination, we applied RNAi-mediated knock down specifically in male germ cells, since Nejire is also expressed in somatic cells of the testis. Knock down of Nejire/dCBP by the germ line limited driver line bamGal4 was efficient in spermatocytes and canoe stage spermatids (Fig 4Bʹ). In *D*. *melanogaster*, several sperm proteins act additively to compact the paternal genome [18]. Therefore, we analysed the expression of ProtA-eGFP, Mst77F-eGFP, Prtl99C-eGFP in the Nejire/dCBP knock down situation.

**Fig 5:**
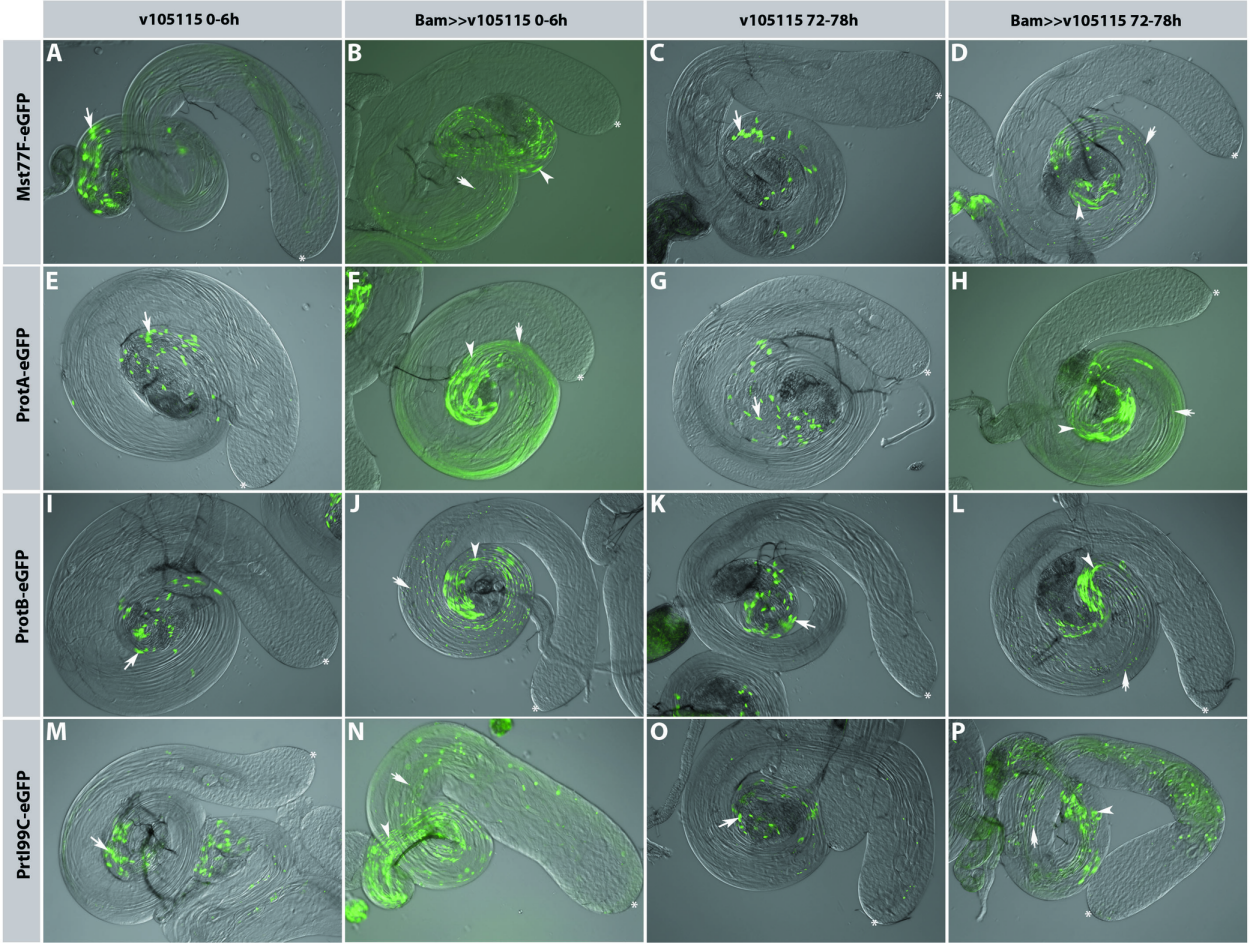
Mst77F, ProtB and Prtl99C are expressed after knock down of Nejire/dCPB. Whole mount preparations (fluorescence and DIG of testes from Mst77F-eGFP (A-D), ProtA-eGFP (E-H), ProtB-eGFP (I-L) and Prtl99C-eGFP (M-P) expressing flies (BamGAl4 strain as the control (A, E, I, M 0–6 h after hatching and C, G, K 72–78 h after hatching), and in the Nejire/dCBP knock down situation (B, F, J, N 0–6 h after hatching and D, G, K, O 72–78 h after hatching). Arrows indicate cysts with elongated spermatid nuclei, arrowheads point to scattered elongated nuclei, double arrows point to scattered small round or oval spermatid nuclei.

In an overview of whole mount testes, it was evident that eGFP-labelled sperm proteins are present in young males (0–6 h after hatching) and three day old males (72–78 h after hatching) (Fig 5). At both stages, the overall morphology of the testes and the presences of elongated flagella were similar in control and Nejire/dCBP knock down males. Spermatid nuclei expressed Mst77F-eGFP (Fig 5A–5D), ProtA-eGFP (Fig 5E–5H), ProtB-eGFP (Fig 5I–5L) and Prtl99C-eGFP (Fig 5M–5P). In the control (bam GAL4 driver line) eGFP-positive nuclei accumulate towards the seminal vesicles, and the synchronously developing spermatid nuclei of cysts were evident (arrow). However, in the Nejire/dCBP knock down situation additional elongated eGFP-positive nuclei (arrowhead) and small round or oval nuclei (double arrow) were scattered towards the hub region (asterisk). In the whole mounts this is particular visible in Fig 5J,5N and 5P. Therefore, we conclude that Nejire is essential for correct maturation of sperm nuclei.

### Post-meiotic *de novo* H3K18 and H3K27 acetylation

We hypothesized that Nejire/dCBP acetylates the histone lysine residues shortly before the time of histone-to-protamine transition. Alternatively, acetylations might persist from the spermatocyte phase and become visible due to the enormous reduction in the nuclear volume [32]. To test this hypothesis of post-meiotic acetylation, we used our pupal testes culture system (as described above). The histone-to-protamine transition takes place between 50–60 h after meiotic divisions [23]. We treated pupal testes of ProtB-mCherry transgenic flies with the HAT inhibitor anacardic acid and the HDAC inhibitor trichostatin A. We chose an incubation time of 24 h to avoid effects of transcriptional regulation in the spermatocyte stage. Thus spermatids at the canoe stage, between 40 h and 60 h after meiosis, went through the spermatocyte stage before we took the testes into culture.

**Fig 6:**
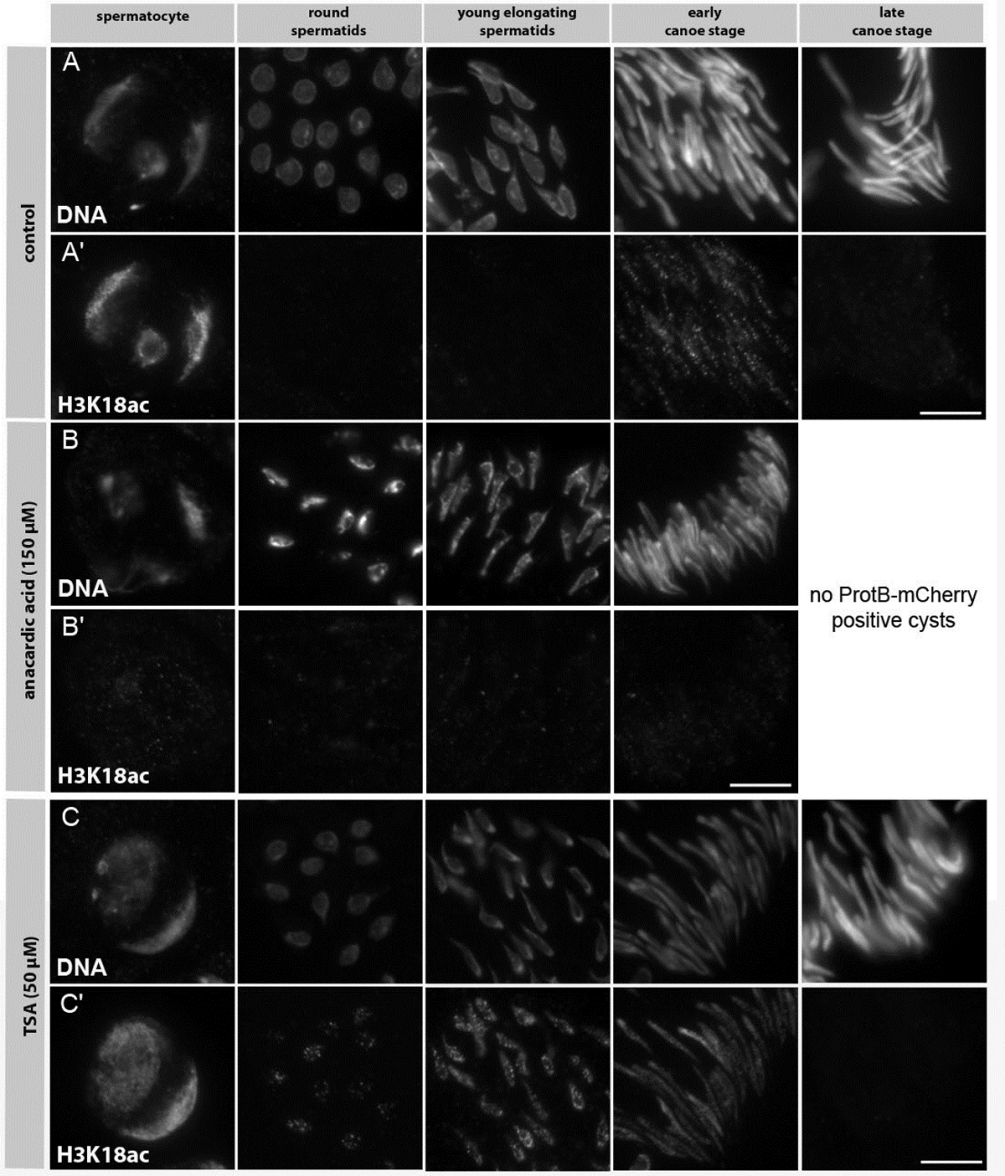
Anacardic acid inhibits post-meiotic acetylation of H3K18. Squashed preparations of spermatid nuclei from pupal testes of ProtB-mCherry expressing flies at 24 h APF incubated for 24 h with either 150 μM AA (B, Bʹ) or 50 μM TSA (C, Cʹ) in comparison to untreated testes (A, Aʹ). (A, B, C) DNA visualized with Hoechst dye. (Aʹ, Bʹ, Cʹ) H3K18ac detected with an anti-H3K18ac antibody. Expression of the ProtB-mCherry fusion protein was used to distinguish early and late canoe stage spermatids (not shown). Treatment with AA leads to a complete absence of H3K18ac signal in post-meiotic spermatid stages (Bʹ, column 4). In comparison to untreated testes (Aʹ, columns 4 and 5) further spermatid differentiation is blocked. In primary spermatocytes H3K18ac was strongly reduced (Bʹ, column 1). Treatment with TSA induced premature H3K18ac appearance in young elongating spermatid nuclei (Cʹ, column 3). TSA also induced increased nuclear H3K18ac levels in primary spermatocytes (Cʹ, column 1). Scale bar: 10 μm.

Immunofluorescence staining with anti-H3K18ac antibody revealed that in contrast to the untreated control testes (Fig 6Aʹ) H3K18ac was almost undetectable in post-meiotic stages in AA-treated testes (Fig 6Bʹ). Testes treated with TSA displayed an increase in the H3K18ac signal (Fig 6Cʹ). Furthermore, H3K18ac was prematurely detected from young elongating spermatid stages onwards (Fig 6Cʹ, columns 3–5). This argues for the activity of a so far uncharacterized enzyme with the same target specificity as Nejire/dCBP. In this experiment differentiating spermatids arrested before or during the histone-to-protamine transition. This might be due to inhibition of more than H3 acetylations in this assay, for example H4 acetylation [23].

Based on these findings we propose that Nejire/dCBP-mediated H3K18 and H3K27 acetylation in spermatids is a specific post-meiotic activity.

### Nejire/dCBP is essential for high levels of *protB* and *prtl99C* transcripts

When monitoring Nejire/dCBP (Fig 4Aʹ) and H3K18ac and H3K27ac (Table 1) during the spermatocyte highly active transcriptional phase, these signals were severely reduced after RNAi-mediated *nejire* knock down (Figs 4Bʹ and 7Bʹ; Panel Bʹ in S1 Fig). *Mst77F*, *protA*, *protB* and *prtl99C* genes are transcribed in spermatocytes and transcripts are under translational repression until the end of the canoe stage. The corresponding proteins accumulate during the late canoe stage in the spermatid nucleus [18, 33]. It is likely that transcription of these genes depends on Nejire/dCBP. To test this, we isolated RNA from testes of the undriven RNAi line v105115 and *bamGAL4* driven RNAi *(v105115)* to quantify *nejire* transcripts. We determined the relative transcript level of *mst77F*, *protA*, *prtl-99C*, *nejire/dCBP* and *tbrd-1* as controls (Fig 8G).

**Fig 7:**
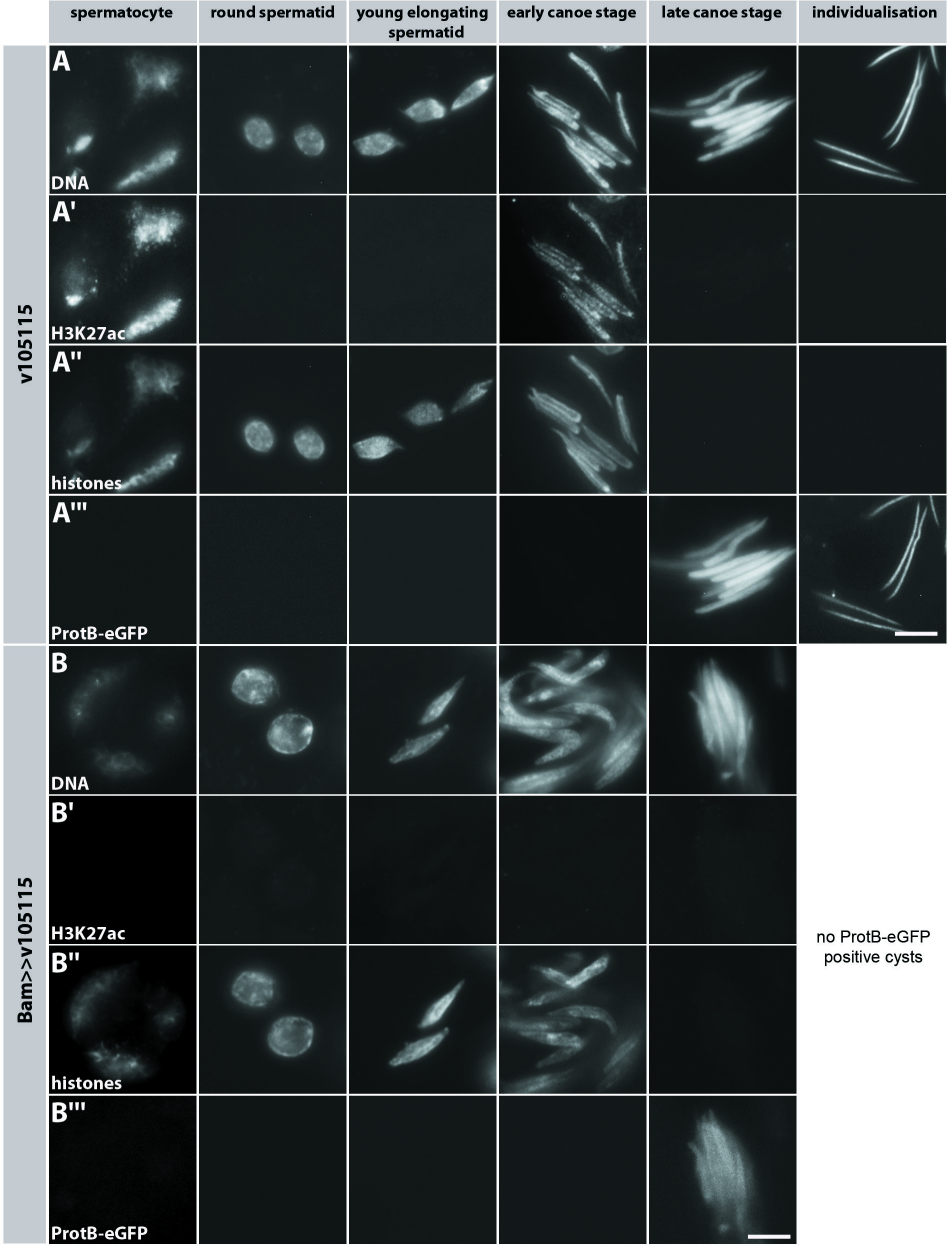
Knocking down Nejire/dCBP *in vivo* results in failure of H3K27 acetylation. Squashed preparations of testes from ProtB-eGFP-expressing wild-type flies (A-Aʹʹʹ) compared to testes of ProtB-eGFP males after Nejire knock down (B-Bʹʹʹ). DNA was visualized with Hoechst dye (A, B). H3K27ac was detected with an anti-H3K27ac antibody (Aʹ, Bʹ); histones were detected with anti-histone-antibody (Aʹʹ, Bʹʹ). ProtB-GFP was used to visualize protamine expression (Aʹʹʹ, Bʹʹʹ). In the wild-type, H3K27ac characterizes the spermatocyte and early canoe stage (Aʹ), histones are detectable until the early canoe stage (Aʹʹ), ProtB-eGFP characterizes nuclei at the late canoe stage and in individualized sperm (Aʹʹʹ). (B) Knock down of Nejire led to a complete absence of individualized sperm and the H3K27 acetylation signal in both spermatocytes and post-meiotic spermatid stages (Bʹ). After *nejire* knock down histones persist until the early canoe stage and degrade as in wild-type (compare Bʹʹ to Aʹʹ). Residual ProtB-eGFP positive spermatid nuclei are observed at the late canoe stage (Bʹʹʹ). Scale bar: 5 µm.

**Fig 8:**
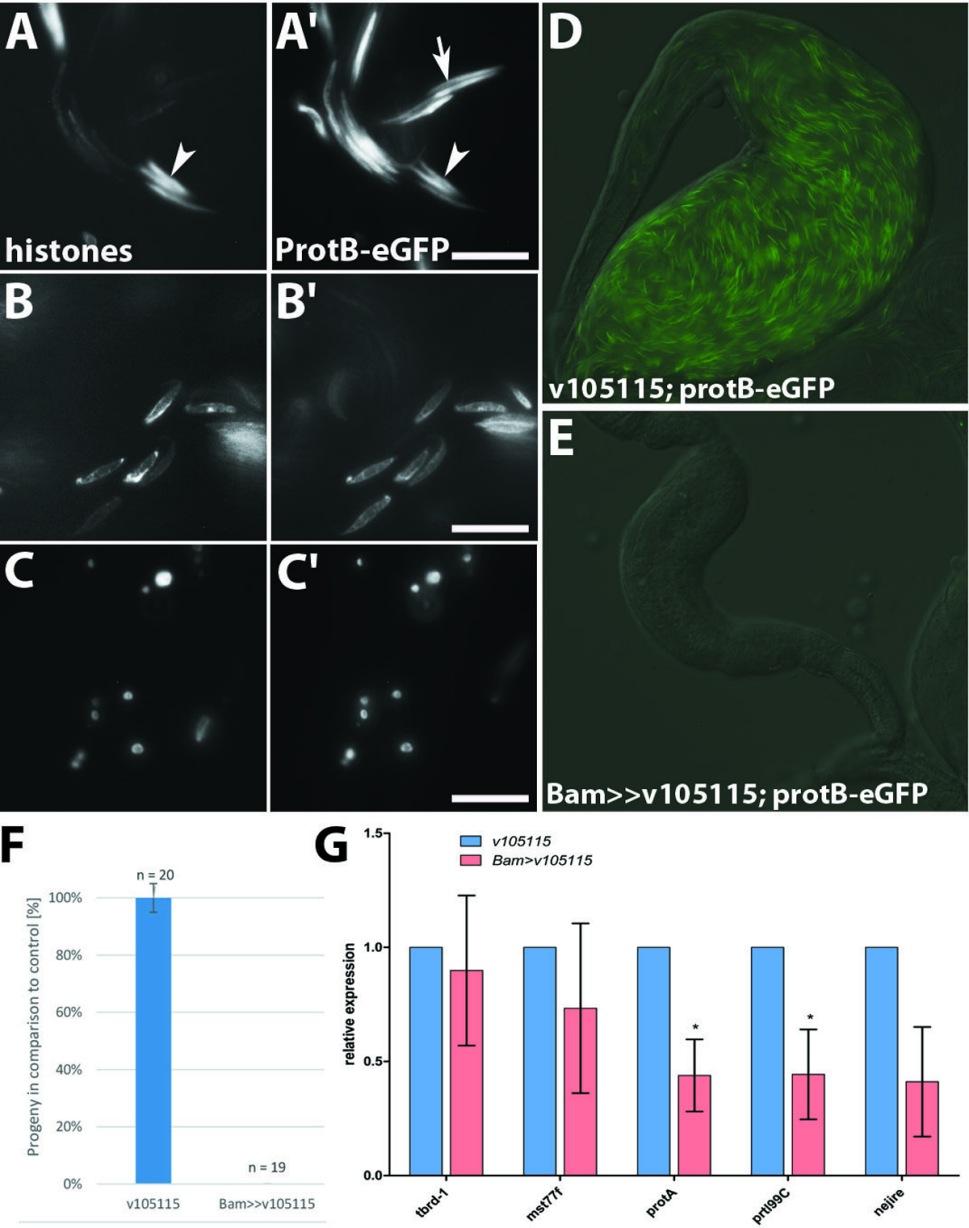
Knock down of Nejire/dCBP reduces transcript levels of ProtB and Prtl99C, and histone degradation, leading to male sterility. Disturbed spermatid nuclei shapes after Nejire/dCBP knock down (A-Cʹ). Distribution of histones was visualized with an antibody against core histones (A-C). ProtB-eGFP distribution in the same nuclei as the core histones (Aʹ-Cʹ, D-E). Elongated spermatid nuclei (A-Aʹ), canoe stage-like nuclei (B-Bʹ), small round and oval nuclei (C-Cʹ). (D-F) Males are sterile after knocking down Nejire/dCBP. Seminal vesicles of v105115 males (control) are filled with ProtB-eGFP positive sperm (D). Seminal vesicles of Bam-GAL4 driven v105115 RNAi males lack ProtB-eGFP positive sperm (E). Bam-GAL4 driven v105115 RNAi males lack progeny (F). *protB* and *prtl99C* transcript levels are significantly reduced after knock down of nejire (* p < 0.05) (G). Scale bar: 5 μm.

In the knock down situation, *nejire* transcripts are reduced considerably. The residual *nejire* transcripts were likely due to Nejire expression in somatic cells of the testes, which are not affected by the *bamGAL4* driver line. tBrd1 transcripts and proteins are limited to the spermatocyte stage [34]. We chose *tbrd1* transcripts since *Mst77F*, *prtl99C*, *protA* and *protB* genes do not belong to tBRD1 target genes [35, 36]. *tbrd-1* transcript levels were not significantly changed upon *nejire* knock down. *Mst77F* transcript levels were not severely reduced in the knock down situation in contrast to *protA* and *prtl99C* transcripts, which were reduced to about 40% of the wild-type level (Fig 8G). We conclude that the transcript levels of *protA* and *prtl99C* depend directly or indirectly on Nejire/dCBP, and thus might arrest spermiogenesis due to insufficient synthesis of sperm chromatin components.

### Nejire/dCBP is essential for efficient post-meiotic histone degradation and male fertility

We subsequently analysed squash preparations for ProtB-eGFP in the Nejire knock down situation with respect to Nejire/dCBP-dependent H3 acetylation, histone degradation and ProtB-eGFP deposition. Here, the characteristic H3K27 (Fig 7Bʹ) and H3K18 (Panel Bʹ in S1 Fig) acetylation pattern was missing in spermatocytes and the early canoe stage. In spermatid bundles in cysts, histones were detectable at the early canoe stage (compare Fig 7Bʹʹ with Fig 7Aʹʹ) but undetectable at the late canoe stage, as in the wild-type situation. However, the spermatid nuclei at the early canoe stage seemed less compact (Fig 7B and 7Bʹʹ) than in wild-type (Fig 7A and 7Aʹʹ).

The phenotypes presented in Fig 7 were most abundant; however, we also observed highly abnormally shaped spermatid nuclei (Fig 8B and 8Cʹ), in agreement with the aberrantly shaped nuclei in whole mount preparations (Fig 5). The shape of ProtB-eGFP positive nuclei was highly variable (Fig 8A–8Cʹ), although similar in the individual nuclei of one cyst. Some protamine positive and histone negative nuclei appeared in this assay (Fig 8Aʹ, arrow). Strikingly, in contrast to wild-type, several slim nuclei contained both histones and ProtB-eGFP (Fig 8A-8Aʹ, arrowhead), a feature not observed in squash preparations of wild-type testes [23]. In particular, we found histones and ProtB-eGFP in early canoe stage-like nuclei (Fig 8B-8Bʹ) and in tid-shaped nuclei (Fig 8C-8Cʹ).

Notably, we found no individualized sperm after Nejire/dCBP knock down in squash preparations (Fig 7). We analysed seminal vesicles of 3-day-old males in the Nejire knock down situation, and again, we could not detect residual sperm in their seminal vesicles (Fig 8E). In contrast, wild type seminal vesicles were full of protamine-eGFP positive sperm (Fig 8D). In agreement with these phenotypes, fertility tests demonstrated that knock down of Nejire led to male sterility (Fig 8F).

In summary, these data indicate that the efficiency of histone degradation and protamination is impaired by Nejire knock down and therefore depends directly or indirectly on H3 acetylation by Nejire/dCBP.

## Discussion

Male germ cells in vertebrates and *Drosophila* undergo a striking compaction of their genome in the haploid phase leading to nearly complete replacement of the histone-based chromatin by protamines. For many years, scientists have been trying to unravel the molecular basis of post-meiotic genome reprogramming that ultimately leads to large-scale genome compaction by the deposition of highly basic DNA-interacting non-histone proteins such as transition proteins and protamines.

### Histone acetylation is a prerequisite for histone crotonylation in early canoe stage spermatids

**Fig 9:**
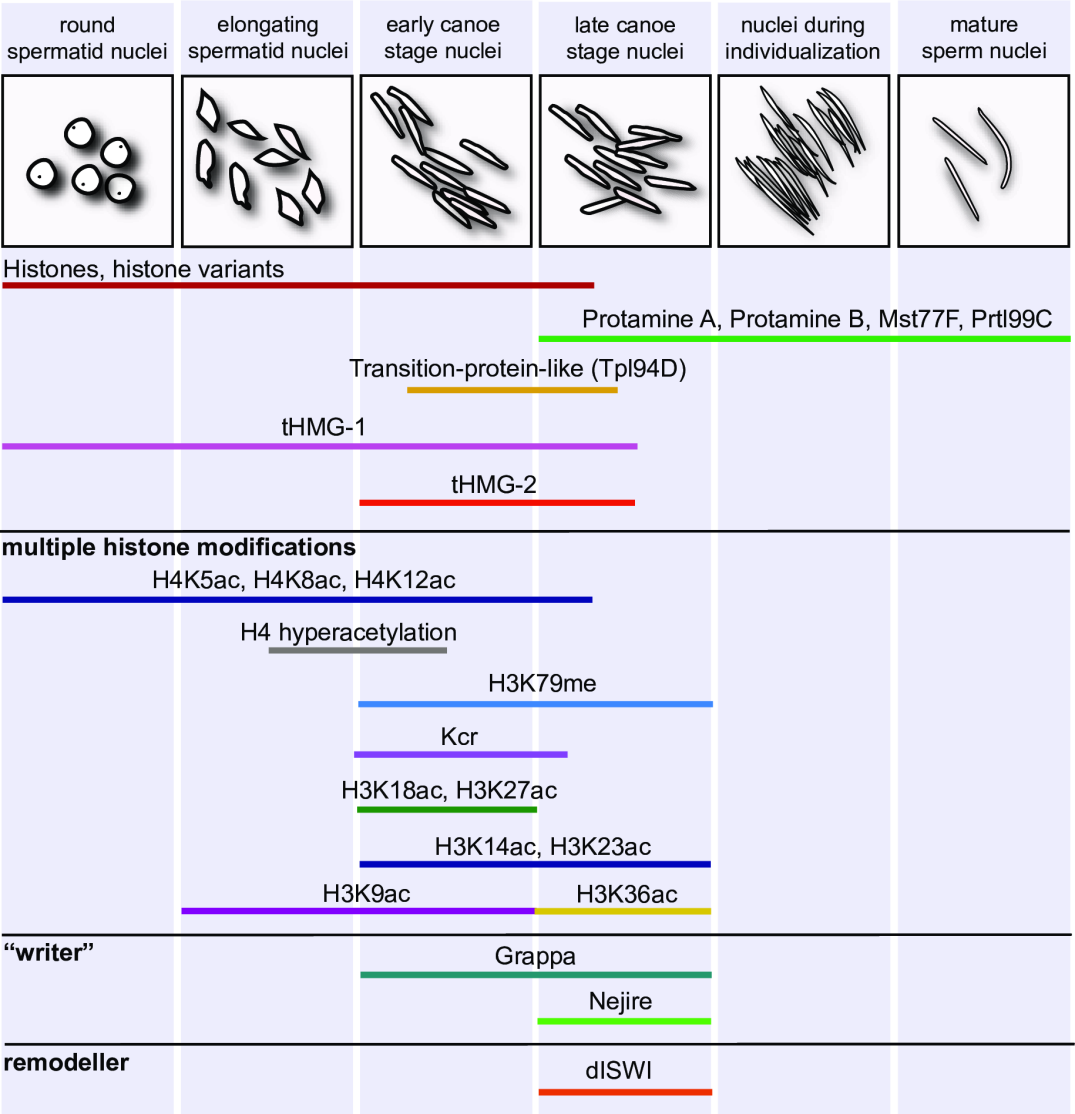
Key chromatin remodelling events and histone modifications in *Drosophila* spermiogenesis. At the top, morphogenesis of the germ cell nuclei is depicted in the order of events after meiosis. Below, histone-to-protamine transition is accompanied by a variety of specific histone modifications associated with Tpl94D, tHMGs, chaperones, Nejire, Grappa and dISWI expression.

A characteristic feature of the post-meiotic chromatin reorganization phase is the deposition of stage-specific histone modifications in flies and mammals. Detailed descriptions of modifications such as acetylation, methylation, phosphorylation, ubiquitination and SUMOylation are available (reviewed in [3, 37]. However, the most striking modification event linked to histone replacement in spermiogenesis is the replication and transcription-independent massive hyper-acetylation of the core histone H4 in early elongating spermatids (Fig 9). Since global histone hyper-acetylation by itself is not enough to mediate removal of histones we tried to identify further modifications playing a putative role in histone-to-protamine transition.

In addition to histone H4 hyper-acetylation and H3K79 methylation [11, 38], we identified lysine crotonylation (Kcr) as another conserved histone modification that precedes removal of histones in the absence of global transcription in *D*. *melanogaster* spermiogenesis (Fig 9). Histone Kcr occurs in a hyper-crotonylation wave in elongating spermatids, coinciding with histone hyper-acetylation in mice [13]. Like histone acetylation and H3K79 methylation, Kcr is a mark of transcriptionally active chromatin in spermatocytes. Using the histone acetyl-transferase inhibitor anacardic acid in pharmacological assays with cultured testes revealed that lysine crotonylation is dependent on post-meiotic histone hyper-acetylation; thus, we assume that crotonylation marks were set *de novo* in spermatids. Since inhibition of acetylation also strongly reduced H3K79 methylation, these modifications likely depend on histone acetylation. These findings indicate that these histone modifications might act in concert to regulate chromatin remodelling during the histone-to-protamine switch.

### A distinct pattern of H3 acetylation characterizes the histone-to-protamine transition phase

In addition, we analysed the distribution of histone H3 acetylation marks in *Drosophila*. In contrast to the conserved H4K5/8/12 acetylation, detectable from spermatocyte stages until histones are removed, H3 lysine acetylation was shown to display a versatile pattern in *Drosophila* spermiogenesis (Fig 9). H3K18, H3K27 and H3K9 acetylation are detectable in stages prior to chromatin remodelling. Here, the observed H3K18 and H3K9 pattern corresponds to the pattern of these acetylation marks in mammals [39]. In *Drosophila* H3K36 acetylation is detectable in late spermatids already loaded with protamines. The vast majority of histones is reported to be replaced by protamines at this stage, but using a specific antibody we showed that at least histone H3 is detectable for longer in canoe stage nuclei than the other core histones [7]. Of interest, a depletion of H3K9 acetylation in mice corresponded to a prolonged retention time of histone H3 on chromatin [12].

A fundamental question is the functional implications of the histone modifications that precede and accompany histone-to-protamine transition. Amalgamating all the stage-specific histone modifications and testis specific histone variants that have been reported, one might generate a testis-specific histone code directing chromatin compaction, histone removal and histone degradation by recruiting a specific machinery acting on modified histones. However, the nature of this histone code remains elusive. In mammals so far, histone hyper-acetylation was hypothesized to lead to an open chromatin structure that facilitates and induces histone displacement.

### Many histone modifications, HMG box proteins, chaperones, Nejire and ISWI characterize the switch from an open nucleosome-based arrangement to highly condensed sperm chromatin

In *D*. *melanogaster*, at least four proteins of sperm chromatin are known: the very similar ProtA and ProtB (also known as Mst35Ba/Bb) and Mst77F and Prtl99C (Fig 9). Biochemical evidence suggests that Mst77F multimerizes to compact chromatin [17]. Mst77F is deposited independently of protamines [40], while protamine deposition seems to depend on the presence of Mst77F [14]. We proposed that these proteins act additively to compact sperm chromatin [18].

Doyen et al [14] suggest that the chromatin remodeller ATPase ISWI is essential for stable incorporation of Mst77F and protamines. Apparently, different chaperones are needed for the loading of these sperm chromatin components [14, 41]. Several different transient HMG proteins (Tpl94D; tHMG-1, tHMG-2) characterize the histone-to-protamine transition phase [7, 42]. Histone modifications (methylation, crotonylation, acetylation) are striking shortly before (early canoe stage) and during the histone-to-protamine transition (late canoe stage).

Using a combination of *in vitro* cultures and inhibitor studies we show that crotonylation depends on previous acetylation, as already known for methylation of H3K79 by Grappa (*Drosophila* Dot1l) [11, 38]. Strikingly, H3K79 methylation and the responsible Dot1-like methyl transferase Grappa characterize the early and late canoe stage [38]. We proposed that Grappa, in cooperation with Rtf1 and UbcD6, marks sites of DNA strand breaks, e.g. by H3K79 methylation, and recruits DNA repair proteins.

Since histone acetylation seemed crucial for progressing to histone-to protamine transition, we aimed to identify acetyl-transferases, in particular those expressed at the canoe stage. H3 acetylation appears later than H4 hyper-acetylation and very specific in the early canoe stage (H3K18ac and H3K27ac). H3K18ac and H3K27ac acetylation marks are set by the aceyl-tranferease Nejire/dCBP.

Here, we present evidence that H3K18 and H3K27 acetylation depends on Nejire. Nejire is essential for fertility and responsible for efficient *protamine* mRNA synthesis. Hyper-acetylation of H3 at K18 and K27 during the histone-to-protamine transition likely is responsible for efficient histone degradation, since inhibition of histone acetyltransferases in the post-meiotic phase abolished H3K18 and H3K27 acetylation. These data are in agreement with *de novo* acetylation by Nejire/dCBP, if the detection in the early canoe stage indeed failed because of accessibility failure for the antibody. Alternatively, we propose that a so far unknown acetyl transferase with the same target specificity exists and that Nejire/dCBP targets other nuclear proteins during the canoe stage. While *in vitro* cultures of testes allowed us to selectively address the consequence of inhibition on post-meiotic stages, RNAi knock down experiments are only possible in the highly transcriptionally active spermatocyte stage. Thus, the distortions seen after knock down of ISWI [14] and Nejire might reflect in part a consequence of their involvement in transcriptional regulation in the spermatocyte phase.

Expression of Nejire during the late canoe stage and the loss of H3K18 and H3K27 acetylation after stage-specific inhibitor application led us to hypothesise that Nejire has dual functions in transcription in spermatocytes and H3 acetylation shortly before and during the histone-to-protamine transition. Nejire/dCBP contains a bromodomain, a protein motif known to specifically bind acetyl-lysine residues (reviewed in [43]. We postulate that Nejire/dCBP is recruited via its bromodomain to already existing histone acetylations and then acetylates H3K18 and H3K27.

### Nejire regulates transcript levels of Prtl99C and protamines as well as efficient histone degradation

Nejire/dCBP is already expressed in spermatocytes, the major transcriptionally active phase ([44] for review see [45]) and later in addition during the phase of protamine loading. This might argue for a dual role of Nejire during spermatogenesis. The knock down of Nejire/dCBP was performed with the male germ line driver line bam*GAL4*, which is active in spermatogonia and spermatocytes. Unfortunately, expression of GAL4 specifically after meiosis is not possible since there is no major transcriptional activity during spermiogenesis. Thus, we cannot interfere with Nejire translation specifically in spermatids. Although knock down of Nejire/dCBP in primary spermatocytes does not lead to obvious defects at this stage, reduced levels of *protamine* mRNA and of *protamine-like 99C* mRNA were observed. This might at least in part explain the observed sterility.

The time selective blocking of acetyl-transferases by anacardic acid after meiosis impedes H3K18 and H3K27 acetylation in late post-meiotic stages. Thus, we hypothesize that these acetylations at this time might contribute to efficient histone-to-protamine transition. Indeed, we observed co-occurrence of histones and ProtB in abnormally shaped spermatid nuclei suggesting inefficient histone degradation. Of interest, depletion of H3K9 acetylation in mice caused a prolonged retention time of histone H3 on chromatin [12].

### *De novo* H3K18 and H3K27 acetylation takes place in the haploid phase, and the corresponding acetyl- transferase Nejire/dCBP is essential for male fertility

Enhanced deposition of post-translational histone modifications in elongating spermatids is suggested to be a consequence of prior events, such as the down-regulation of enzymes involved in their removal from histones, rather than the presence of specific enzymes generating these modifications (reviewed in [5]). Here, we show that in particular H3K18 and H3K27 acetylations appear *de novo* during the preparation and implementation of the histone-to-protamine transition during the canoe stage. Strikingly, these modifications depend directly or indirectly on the histone acetyl-transferase Nejire/dCBP. In mice, a partially depleted version of CBP led to fertile male mice [46]. In contrast, the efficient reduction of Nejire/dCBP by RNAi in male germ cells of *Drosophila* led to male sterility, underscoring the importance of this acetyl-transferase for spermatogenesis.

## Conclusions

In our view, Nejire likely regulates H3K18 and H3K27 acetylation during transcription in spermatocytes. H3K18 and H3K27 are prominent also during the histone-protamine transition. We hypothesise that male sterility is directly and indirectly dependent on Nejire and caused by failure H3K18 and H3K27 acetylation in both stages.

In conclusion, with respect to the histone-to-protamine transition we propose that Nejire regulates transcript levels for sperm relevant proteins in spermatocytes and in directly or indirectly acetylation of H3K18 and H3K27 for fully efficient histone degradation. We propose that the numerous different histone modifications are required for histone degradation.

## Supporting information

S1 Fig Knocking down Nejire/dCBP *in vivo* results in failure of H3K18 acetylation. Squashed preparations of testes from ProtB-eGFP expressing wild-type flies (A-Aʹʹ) in comparison to testes of ProtB-eGFP males after Nejire/dCBP knock down (B-Bʹʹ). DNA was visualized with Hoechst dye (A, B). (Aʹ, Bʹ) H3K18ac detected with an anti-H3K18ac antibody, (Aʹʹ, Bʹʹ) ProtB-GFP was used to visualize protamine expression. In the wild-type, H3K18ac characterizes the spermatocyte and early canoe stage (Aʹ), ProtB-eGFP characterizes nuclei at the late canoe stage and in individualized sperm (Aʹʹ), Knock down of Nejire/dCBP led to a complete absence of individualized sperm (B-Bʹʹ) and the H3K27 acetylation signal in spermatocytes and in post-meiotic spermatid stages (Bʹ). After Nejire/dCBP knock down, residual ProtB-eGFP positive spermatid nuclei are observed (Bʹʹ). Scale bar: 5 µm.
(JPG)

S1 Table Acetyl transferases with predicted expression in the *Drosophila* testis. (DOCX)
