# Supplementary figures and images for: Nejire/dCBP-mediated histone H3 acetylation during spermatogenesis is essential for male fertility in Drosophila melanogaster
Source: PLoS One. 2018 Sep 7;13(9):e0203622. doi: 10.1371/journal.pone.0203622 (PMC6128621; doi:10.1371/journal.pone.0203622)

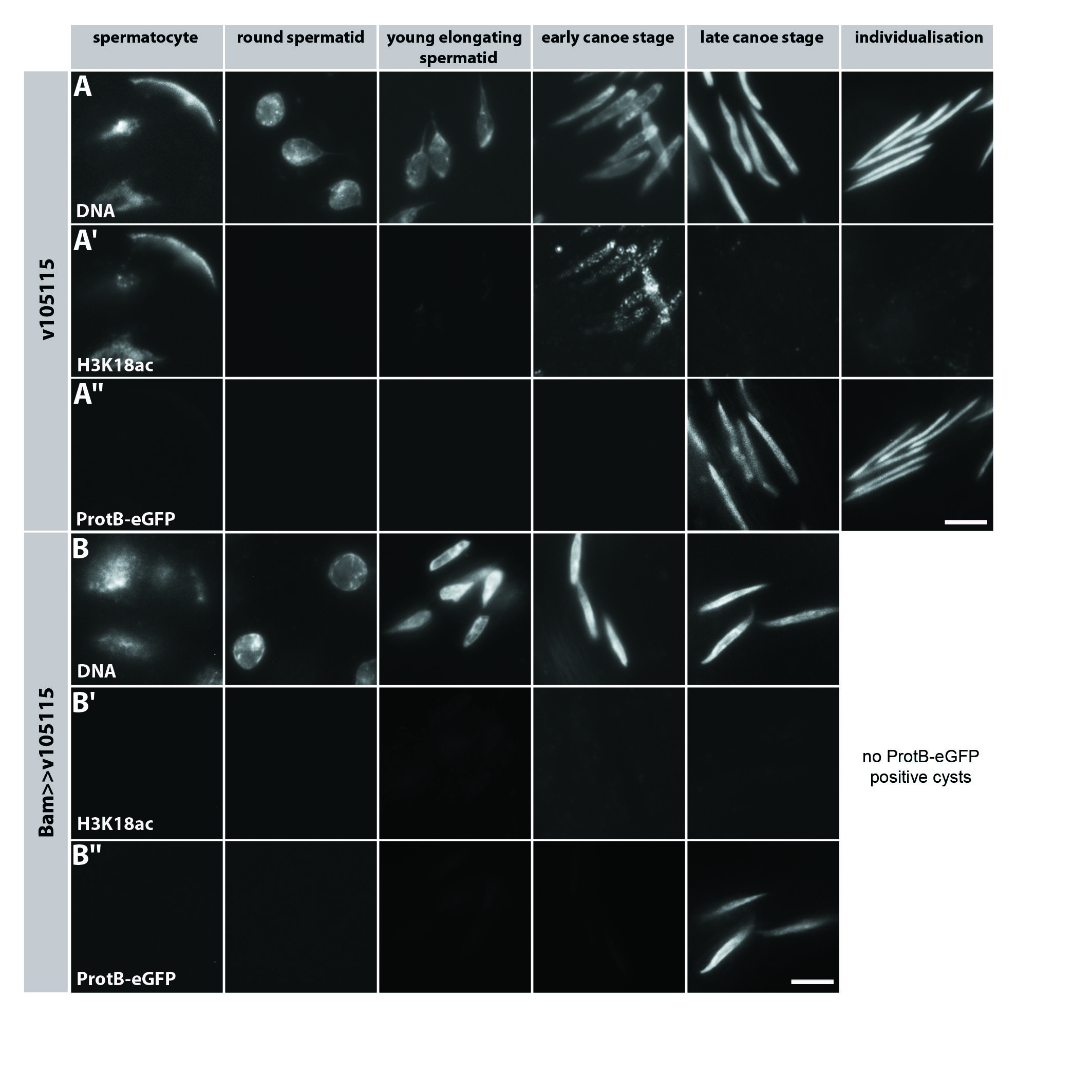

Supplement: S1 Fig — Squashed preparations of testes from ProtB-eGFP expressing wild-type flies (A-Aʹʹ) in comparison to testes of ProtB-eGFP males after Nejire/dCBP knock down (B-Bʹʹ). DNA was visualized with Hoechst dye (A, B). (Aʹ, Bʹ) H3K18ac detected with an anti-H3K18ac antibody, (Aʹʹ, Bʹʹ) ProtB-GFP was used to visualize protamine expression. In the wild-type, H3K18ac characterizes the spermatocyte and early canoe stage (Aʹ), ProtB-eGFP characterizes nuclei at the late canoe stage and in individualized sperm (Aʹʹ), Knock down of Nejire/dCBP led to a complete absence of individualized sperm (B-Bʹʹ) and the H3K27 acetylation signal in spermatocytes and in post-meiotic spermatid stages (Bʹ). After Nejire/dCBP knock down, residual ProtB-eGFP positive spermatid nuclei are observed (Bʹʹ). Scale bar: 5 µm. (JPG) [file pone.0203622.s001.jpg]
