# Supplementary material for: Nejire/dCBP-mediated histone H3 acetylation during spermatogenesis is essential for male fertility in Drosophila melanogaster
Source: PLoS One. 2018 Sep 7;13(9):e0203622. doi: 10.1371/journal.pone.0203622 (PMC6128621; doi:10.1371/journal.pone.0203622)
Supplement: S1 Table — (DOCX) [file pone.0203622.s002.docx]

| **Protein** | **acetyl transferase family** | **known**  **target** | **transcript in testis**  **(FlyAtlas)** | **other remarks** |
| --- | --- | --- | --- | --- |
| Nej | P300/CBP | H3K18; H3K27 | low |  |
| Mof | MYST | H4K16 | low | own results: spermatocytes |
| Chm | MYST | H3, H4 | low |  |
| Tip60 | MYST |  | low |  |
| CG1894 | MYST | histones | moderate |  |
| Enok | MYST | H3K23 | low |  |
| dGCN5/PCAF | GNAT | H3, H4 | low |  |
| San | GNAT |  | moderate |  |
| AgmNAT | GNAT |  | none | nitrogin metabolism |
| Naa20A | GNAT |  | low |  |
| CG15628 | GNAT |  | low |  |
| AANATL2 | GNAT |  | none | nitrogin metabolism |
| CG12560 | GNAT |  | none |  |
| CG17681 | GNAT |  | none |  |
| CG15155 |  |  | none |  |
| CG5783 | GNAT |  | low |  |
| Atac2 | GNAT | H2A, H4K16 | low |  |
| Eco | GNAT | histones | low | mitotic sister chromatin cohesion |
| Naa60 | GNAT | histones | low | Nucleosome assembly, chromosome segregation, Golgi |
| Vnc/Ard1 | GNAT | histones | high  (RNAseq) | female germ line  mutants: female sterile |
| CG8481 | GNAT |  | moderate | mutants: fertile |
| CG4210 | GNAT |  | none |  |
| Naa40 | GNAT |  | moderate | mutants: fertile |
| Gnpnat | GNAT |  | low  (RNAseq) | UDP-N-acetylglucosamine biosynthetic process |
| CG11539 | GNAT | histones | low |  |
| CG31730 | GNAT |  | high |  |
| Naa20B | GNAT |  | moderate | mutants fertile |
| Naa30B | GNAT |  | low |  |
| Ada3 |  | H4K12; H3K9 | low |  |
| Hat1 |  | H4 | moderate |  |
| L(1)G0020 | GNAT |  | none | tRNA  N-acetyltransferase activity |
| Naa30A | GNAT |  | low |  |

**S1 Table. Acetyl transferases with predicted expression in the *Drosophila* testis**
